# Transformational machine learning: Learning how to learn from many related scientific problems

**DOI:** 10.1073/pnas.2108013118

**Published:** 2021-11-29

**Authors:** Ivan Olier, Oghenejokpeme I. Orhobor, Tirtharaj Dash, Andy M. Davis, Larisa N. Soldatova, Joaquin Vanschoren, Ross D. King

**Affiliations:** ^a^School of Computer Science and Mathematics, Liverpool John Moores University, Liverpool L3 5UX, United Kingdom;; ^b^Department of Chemical Engineering and Biotechnology, University of Cambridge, Cambridge CB3 0AS, United Kingdom;; ^c^Anuradha and Prashanth Palakurthi Centre for Artificial Intelligence Research, Department of Computer Science & Information Systems, Birla Institute of Technology and Science Pilani, Goa 403726, India;; ^d^Discovery Sciences, Biopharmaceuticals R&D, AstraZeneca, Cambridge CB4 0WG, United Kingdom;; ^e^Department of Computing, Goldsmiths, University of London, London SE14 6NW, United Kingdom;; ^f^Department of Mathematics and Computer Science, Eindhoven University of Technology 5612 AZ Eindhoven, The Netherlands;; ^g^Alan Turing Institute, London NW1 2DB, United Kingdom;; ^h^Department of Biology and Biological Engineering, Chalmers University of Technology SE-412 96 Göteborg, Sweden

**Keywords:** AI, drug design, transfer learning, stacking, multitask learning

## Abstract

Almost all machine learning (ML) is based on representing examples using intrinsic features. When there are multiple related ML problems (tasks), it is possible to transform these features into extrinsic features by first training ML models on other tasks and letting them each make predictions for each example of the new task, yielding a novel representation. We call this transformational ML (TML). TML is very closely related to, and synergistic with, transfer learning, multitask learning, and stacking. TML is applicable to improving any nonlinear ML method. We tested TML using the most important classes of nonlinear ML: random forests, gradient boosting machines, support vector machines, k-nearest neighbors, and neural networks. To ensure the generality and robustness of the evaluation, we utilized thousands of ML problems from three scientific domains: drug design, predicting gene expression, and ML algorithm selection. We found that TML significantly improved the predictive performance of all the ML methods in all the domains (4 to 50% average improvements) and that TML features generally outperformed intrinsic features. Use of TML also enhances scientific understanding through explainable ML. In drug design, we found that TML provided insight into drug target specificity, the relationships between drugs, and the relationships between target proteins. TML leads to an ecosystem-based approach to ML, where new tasks, examples, predictions, and so on synergistically interact to improve performance. To contribute to this ecosystem, all our data, code, and our ∼50,000 ML models have been fully annotated with metadata, linked, and openly published using Findability, Accessibility, Interoperability, and Reusability principles (∼100 Gbytes).

## Significance

Machine learning (ML) is the branch of artificial intelligence (AI) that develops computational systems that learn from experience. In supervised ML, the ML system generalizes from labelled examples to learn a model that can predict the labels of unseen examples. Examples are generally represented using features that directly describe the examples. For instance, in drug design, ML uses features that describe molecular shape and so on. In cases where there are multiple related ML problems, it is possible to use a different type of feature: predictions made about the examples by ML models learned on other problems. We call this transformational ML. We show that this results in better predictions and improved understanding when applied to scientific problems.

Machine learning (ML) develops computational systems that learn from experience (1–4). ML has a long history of application to science, one of the first ML programs being Meta-Dendral, which used ML to improve the analysis of mass-spectrometric data (5). The importance of ML to science is now widely recognized, and ML is now being applied to almost all areas of science: drug discovery (6), organic synthesis planning (7), materials science (8), medicine (9), and so on.

Most ML represents examples using tuples of attributes, i.e., the data can be put into a single table, with the examples as rows and the attributes as columns (1–4). Attributes are features of examples which are believed to be important. Currently, such features are almost always intrinsic properties. For example, if one wished to learn about the pharmacological activity of a drug, then properties of its molecular structure would be useful attributes. Typically, one attribute is singled out for prediction, and the other attributes contribute information to make this prediction. If the predicted attribute is categorical then the problem is a discrimination/classification task, and if the attribute is a real number then the problem is a regression one. Here we focus on regression.

In cases where there are multiple related ML problems (tasks) it is possible to use extrinsic features: predictions made about examples by ML models learned on other tasks. We call this transformational ML (TML). TML transforms a representation based on intrinsic attributes of examples to an extrinsic representation based on the predictions of previously learned models. As we will discuss, TML is very closely related to and synergistic with stacking, multitask learning (MTL), and transfer learning (TL). It enables the utilization of knowledge previously learned from related tasks, rather than learning each new model from scratch. TML is thus a metalearning idea that is applicable to enhancing any nonlinear ML method. It is particularly well suited when there exist many related small learning tasks.

**Fig. 1.**
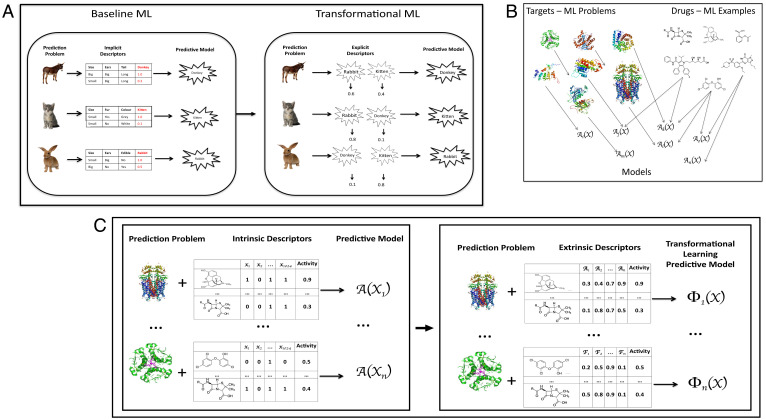
(*A*) Baseline ML vs. TML for prediction of animals. We illustrate TML using a toy example with three ML related problems: donkey, kitten, and rabbit prediction. In baseline ML intrinsic attributes (descriptors) are used to build predictive models, e.g., *size, ears, edible.* Instantiation of these attributes in the rabbit example gives: *Rabbit*(*Size = Small, Ears = Big, and Edible = No*) *→* 1.0. *Rabbit*() is a mathematical model that inputs instantiated examples of animals and outputs the probability of an example’s being a rabbit. For TML three baseline models are learned: *Donkey*(), *Rabbit*(), and *Kitten*(). The predictions from these baseline models are used as extrinsic attributes for TML learning. Using the same rabbit training example (*Size = Small, Ears = Big, and Edible = No*) produces the TML instantiation Rabbit (Donkey (Size=Small,Ears=Big, _),Kitten (Size=Small,Ears=Big, _))→1.0, which evaluates to give the TML example: *Rabbit*(*0.1, 0.8, _*) *→ 1.0.* Intuitively, one can see the TML form of representation is useful because a rabbit has some resemblance to a donkey in having cute long ears and to a kitten in being small and cute. Note that this transformed representation enables TML models to capture attributes of animals not originally used, like cuteness, having eyes at the side of the head (shared by rabbits and donkeys), and so on. (*B*) Learning QSAR models. The QSAR prediction task is as follows: Given a target (usually a protein) and a set of chemical compounds (small molecules) with associated activities (e.g., inhibition of the target protein), learn a predictive mapping from molecular representation to activity. The ML problems (tasks *T_i_, i = 1,…, n*) are characterized by a target protein, together with a set of drugs with associated activities [*X_i_ =* (*x_1_, x_2_, …, x_p_*)]. The learned ML models are represented as *A_i_*(*X_i_*)*.* (*C*) Baseline ML vs. TML for QSAR prediction. In applying baseline (standard) ML to QSAR problems, each target protein is associated with multiple drugs each of which is described by intrinsic attributes, in this case Boolean values indicating the presence or absence of specified chemical groups. These attributes along with associated activities are used to learn a model *A_i_*(*X_i_*) *→ activity.* The examples are of the form *A_i_*(*1, 0, 1, 1, …*) *→ 0.9*. In applying TML to QSAR problems the attributes are now the predictions from baseline QSAR models *A_i_*. The examples are therefore of the form $\Phi_{1}(A_{2}\left(1,\;0,\;1,\;1,\;\ldots \right),A_{3}\left(1,\;0,\;1,\;1,\;\ldots \right),\;\ldots )\to 0.9.$ Note that model *Φi* only includes predictions for the other tasks. This produces the following example after running the baseline ML QSAR models: $\Phi_{1}\left(0.2,\;0.3,\;\ldots \right)\to 0.9.$

To intuitively explain this idea, we take as an illustrative example the problem of learning to recognize multiple animal species (Fig. 1*A*). If there are many types of animals, with new ones expected to be added, then it would be reasonable to learn classifiers for each species, rather than learning a single large classifier. The standard (baseline) ML approach to such a learning task would be to use intrinsic attributes (e.g., size or presence of fur) to learn these prediction models. The TML approach is to first learn prediction models for all known species in the standard way and then to use the predictions from these learned baseline models to represent all animals, i.e., by their “catness,” “rabbitness,” or “horseness,” and to train a (meta) ML model (Fig. 1*A*) to make predictions using this representation. TML is applicable to any domain where ML tasks share a common set of intrinsic features and related target variables, which is commonly the case in scientific domains, e.g., drug design where targets (proteins) can be related, as can drugs (Fig. 1*B*). The underpinning justification for TML is utilization of prior knowledge about the regularity of the world encoded in the previously learned prediction models.

More formally, the input to TML is a set of previously learned prediction models and a new learning task with labeled examples. TML is performed in two stages. First, the examples from the new learning task are applied to the prediction models and the predictions of the models used to generate the transformed representation. Then, the transformed representation is used to learn a prediction model for the new task (Fig. 1). Consider a set of *n* tasks (learning problems) *T_i_, i = 1.n*, each represented by a common set of *p* attributes *X_i_ =* (*x_1_*, *x_2_*, …, *x_p_*), and a unique prediction attribute *y_i_*. On each task we train a model using a baseline ML method *A*, yielding *n* models *A_i_* = *A*(*X_i_*) ≈ *y_i_.* We then apply an ML method *Φ* (possibly different from *A*) to predict a new target *y_new_* for a new task *T_new_* by using the *n* previously trained models *A_i_* to generate *n* latent features *y_i_* and learning the relationship from the latent features to the new target *y_new_*: *Φ*(*X_new_*) = *Φ*(*A_1_* (*X_new_*), *A_2_* (*X_new_*), …, *A_n_* (*X_new_*)) = *Φ*(*y_1_*, *y_2_*, …, *y_n_*) ≈ *y_new_*.

In the case of QSAR (quantitative structure activity relationships) predictions, a common step in early-phase drug discovery (23, 24), the task *T_i_,* is as follows: given a target (usually a protein) and a set of chemical compounds (small molecules) with associated activities (e.g., inhibition of the target protein), learn a predictive mapping from molecular representation to activity: *X_i_* is a set of drug descriptors with known activity *y_i_* (Fig. 1*B*). Baseline ML methods (e.g., random forest and k-nearest neighbor [k-NN]) are first applied to each QSAR prediction task *T_i_,* yielding prediction models *A_i_ → activity* [of the form *A_i_*(*1, 0, 1, 1, …*) *→ 0.9*; see Fig. 1*C*]. In the TML approach, for a new QSAR task we apply an ML method *Φ* (which could be different from any of previously used *A_i_*, or one previously used) to make a new prediction by using the *n* previously trained models *A*(*X_i_*). The attributes are now the predictions from baseline QSAR models *A_i_* (of the form *Φ*(*0.2, 0.3, …*) *→ 0.9*; see Fig. 1*C*).

TML has very close similarities to other ML approaches. However, the specific TML concept does not seem to have been previously identified or systematically evaluated. TML has very close similarities to MTL (10). In MTL related problems (tasks) are learned simultaneously with the aim of exploiting similarities between the problems to improve performance. MTL has been successful in many scientific applications (e.g., refs. 11 and 12). MTL is defined as “an approach to inductive transfer that improves generalization by using the domain information contained in the training signals of related tasks as an inductive bias. It does this by learning tasks in parallel while using a shared representation; what is learned for each task can help other tasks be learned better” (10). MTL starts, as does TML, with a set of *n* task (learning problems) *T_i_, i = 1…n,* each represented by a common set of *p* attributes *X_i_ =* (*x_1_, x_2_,…, x_p_*), and a unique prediction attribute *y_i_*. MTL aims to improve the learning of a model for *T_i_* using ML method *A* by learning in parallel all the *n* models *A_i_ = A*(*X_i_*) *= y_i_* (12). There are two main differences between MTL and TML: MTL typically learns the tasks in parallel, while TML typically learns tasks sequentially, and in TML information is shared between tasks using the data representation, while MTL uses a single model.

TML is also very closely related to TL (13), where information is transferred from a specific source problem to a specific target problem. The idea of TL is to extract knowledge from one or more source domains and to reuse this knowledge in a target domain where data are scarce, with the aim of building better-performing learning models in the target domain. Lin and Jung (14) define TL as “given a source domain *D_S_* and learning task *T_S_*, and a target domain *D_T_* and learning task *T_T_*, TL aims to help improve the learning of the target predictive function *f*(*•*) in *D_T_* using the knowledge in *D_S_* and *T_S_*, where *D_S_ ≠ D_T_,* or *T_S_ ≠ T_T_*” (14). This definition of TL is very general but typically TL differs from TML in that just one source task is learned, while TML requires many source tasks. TL has previously been successfully applied in drug design with several prospective applications demonstrating the usefulness of this ML approach (15).

TML resembles MTL in using a single joint representation and TL and metalearning in transferring task information. However, in TML instead of using a predefined similarity measure or another criterion to preselect a set of similar tasks the different tasks are projected into a joint numeric representation (embedding), and then any ML can be applied to this new transformed representation to learn how to make accurate predictions for a specific problem.

TML also has very close similarities to stacking (16, 17), a form of ensemble ML. In ensemble ML multiple learning methods are combined to obtain better predictive performance than could be obtained from any of the constituent learning algorithms alone. In stacking multiple baseline models are first learned, then a metamodel is learned using the outputs of the baseline level model. Stacking starts with a single task *T_i_*, represented by a set of *p* attributes *X_i_ =* (*x_1_, x_2_,…, x_p_*), and a unique prediction attribute *y_i_*. We then train m baseline models using m baseline ML methods *A_j_, j = 1…m. A_j_*(*X_i_*) *= y_ij_.* We then apply a ML method Φ (possibly different from any *A_j_*) to learn the relationship between the latent features *y_ij_* and *y_i_*. The main difference between TML and stacking is that TML learns across a large set of tasks T_i_, i = 1…n, each containing potentially different examples, while in stacking different baseline models are typically trained on the same task; e.g., one might stack together random-forest and neural-network predictors for a specific problem. In contrast, in TML the models are not trained on the same task and could simply be a set of pretrained models.

**Fig. 2.**
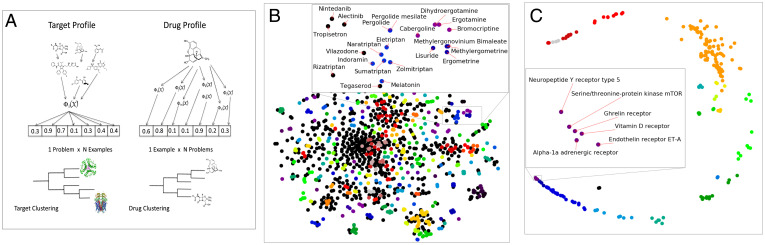
(*A*) Clustering methodology. To cluster the drugs (examples) we applied the TML models to form example profiles. In these profiles each element is the predicted value of the drug on one of the targets (problems). For example, in the case of penicillin the first element could be its predicted activity from the *H. sapiens* serotonin 5a receptor model, the second element its predicted activity on the *M. musculus* DHFR model, and so on. Using the n example profiles, the drugs are clustered using hierarchical clustering (see *SI Appendix*, *Clustering*). To cluster the targets (problems) we first used the TML models to form problem profiles, one profile for each problem. In a problem profile each element is the predicted value of a specific drug (example) on the target (problem). For example, in the case of the QSAR prediction problem *H. sapiens* DHFR, the first element could be the predicted activity of triclosan, the second of penicillin, and so on. To form the final clustering we utilized the n prediction profiles and then clustered them using hierarchical clustering (see *SI Appendix*, *Clustering*). (*B*) Chemical compounds clustered by activity on QSAR targets. The figure shows the overall clustering of FDA-approved compounds (colors representing clusters) and a magnified section of the clustering with three closely related clusters and singletons. The magnified section includes compounds treating migraines, pituitary tumors, bleeding during pregnancy, Parkinson's disease, hypertension, sleep disorders, depression, irritable bowel syndrome, nonsmall-cell lung cancers, and so forth. The TML clustering reveals order to this complexity. All compounds are structurally either ergot-derived (clusters 1 and 2) and tryptamines (cluster 3)—pergolide is the closest compound in cluster 3 to clusters 1 and 2 and is an exception in being ergot-derived. The serotonin link occurs even when the primary pharmacology is different. For example, indoramin is classed as antiadrenergic agent, but it is also a serotonin receptor inhibitor (43); alectinib is primary an anaplastic lymphoma kinase inhibitor, but it also a serotonin receptor inhibitor (44). Many of the compounds in cluster 3 are used to treat migraines (eletriptan, naratriptan, sumatriptan, and zolmitriptan). It is insightful that the other two remaining compounds in the cluster are also effective against migraines: indoramin (45) and melatonin (46). Below melatonin in the clustering is tegaserod, which was used to treat irritable bowel syndrome (IBS constipation). It is noteworthy that melatonin is also effective against IBS (47). Tegaserod was withdrawn by the FDA because of increased risks of heart attack or stroke. The position of tegaserod as an outlier to a cluster of compounds involved in vasoconstriction might have provided a warning of this problem. (*C*) Drug targets clustered by chemical profile. The figure shows the overall clustering of the protein targets of FDA-approved drugs (colors representing clusters) and a magnified section of one cluster (see *SI Appendix*, *Clustering*). The proteins in the cluster do not share any obvious structural similarity: mTOR is a serine/threonine-protein kinase; vitamin D receptor is a nuclear receptor transcription factors; and Endothelin receptor ET-A, Neuropeptide Y receptor type 5, Ghrelin receptor, Alpha-1a adrenergic receptor, Corticotropin releasing factor receptor 2, and Melanocortin receptor 3 are all G protein-coupled receptors. These proteins in the cluster are also not currently known to be linked by disease or biochemical pathway. However, there is a clear theme to the function of these (mammalian) proteins, related to control of metabolism: the mTOR pathway is a central regulator of mammalian metabolism and physiology; ghrelin is the “hunger hormone”; vitamin D is involved in controlling growth; neuropeptide Y is associated with control of food intake; the alpha-1a adrenergic receptor is associated with the flight-or-fight response; corticotropin releasing factor affects aggression, feeding, and locomotor activity; and the melanocortin system is thought to play a fundamental role in the control of feeding and body weight. This interpretation is supported by the growth hormone-releasing hormone receptor clustering nearby.

Within the field of drug design TML also closely resembles the idea of using ML models to predict affinity fingerprints (18). Similarly, in natural language processing, Strubell et al. (19) have successfully used a MTL/TL approach that resembles TML.

TML also resembles the concept of an inductive database (ID) (20) in its focus on multiple models. An ID is a general-purpose database in which both the data and ML models can be represented, retrieved, and manipulated. TML is similar in its focus in considering ML models to objects of interest outside of their initial purpose. It differs in being directly focused on a specific method of using models to aid prediction.

TML is applicable to improving any nonlinear ML method. To evaluate TML we selected five ML methods that exemplify the main families of nonlinear ML methods (1–4): random forests (RF) (21), gradient-boosting machines (XGB) (22), support-vector machines (SVMs) (23), k-NN (3), and neural networks (NN) (3, 4). To ensure the generality and robustness of the evaluation we utilized thousands of ML problems from three important scientific problems: drug discovery (QSAR learning), predicting human gene expression (across different tissue types and drug treatments), and metamachine learning (predicting how well ML methods will work on problems). For each ML method, and each problem area, we compared TML vs. baseline (standard) ML. We investigated two forms of prediction improvement: strong improvement, where predictions made using the new TML features outperform those based on the baseline (intrinsic) features [*Φ*(*X_TML_*) vs. *Φ*(*X_baseline_*)] and combined improvement, where the new TML features improve performance through augmenting the baseline features [*Φ*(*X_TML_* plus *X_baseline_*) vs. *Φ*(*X_baseline_*)]. To augment the TML predictions we used the simplest possible form of stacking: combining the predicted outputs. We found that TML significantly improved the average predictive performance of all methods in all three domains (from 4 to 50%), i.e., models trained on the novel extrinsic features generally outperformed those trained on the intrinsic ones (Table 1).

**Table 1.** Prediction results

Problem area	ML method	Baseline	Transformed	Stacked: convex	Stacked: ridge	Significance: sign	Significance: Wilcoxon
QSAR	RF	0.6647	**0.6609**	0.6616	0.6491	<0.0001	<0.0003
	XGB	0.6944	0.6590	0.6809	**0.6462**	<0.0001	<0.0001
	SVM	0.6735	0.6696	**0.6619**	0.6700	<0.0001	<0.0001
	KNN	0.7158	0.7360	0.6989	**0.6982**	<0.0001	<0.0001
	NN	0.6638	**0.6494**	0.6761	0.6658	<0.0001	<0.0001
LINCS	RF	0.0694	**0.0664**	0.0664	0.0670	<0.0001	<0.0001
	XGB	0.0687	**0.0669**	0.0669	0.0688	<0.0001	<0.0001
	SVM	0.0692	**0.0677**	0.0692	0.0687	<0.0001	<0.0001
	KNN	0.0719	0.0721	0.0715	**0.0689**	<0.0001	<0.0001
	NN	0.0742	0.0707	0.0715	**0.0703**	<0.0001	<0.0001
Meta-ML	RF	0.1203	0.0607	**0.0605**	0.0792	<0.0001	<0.0001
	XGB	0.1268	**0.0718**	0.0738	0.0874	<0.0001	<0.0001
	SVM	0.1400	**0.1007**	0.1008	0.1111	<0.0001	<0.0001
	KNN	0.1445	0.1277	**0.1273**	0.1274	<0.0001	<0.0001
	NN	0.1320	**0.1096**	0.1105	0.1104	<0.0001	<0.0001

All results are average root-mean-squared error (RMSE). Results underlined are the best for an application area. Results in bold are the best for an ML method on an application area. The baseline results are for the tuned standard ML methods using standard intrinsic representations. The transformed results are the TML results using the extrinsic representations. The results are averaged over the thousands of problems in the different application areas. We tested two forms of stacking: convex (nonnegative least) squares and ridge (ridge regression). We used two forms of significance test: sign (sign test) and Wilcoxon (Wilcoxon signed-rank test). Both check for RMSE inequality (*P* value < 0.05) between standard and transformed methods; the former tests whether their RMSE medians are statistically different, while the latter tests whether their RMSE means are different.

Almost every form of statistical and ML method has been applied to QSAR learning (23), but no single method has been found to be clearly best (24, 25). QSAR problems are well-suited to TML as they can be related by having related target proteins (e.g., the problem of inhibiting the enzyme dihydrofolate reductase [DHFR] in *Mus musculus* [mouse] and *Homo sapiens* are similar because they have similar ligand binding sites [active centers]) (26), and they involve the same or chemically related molecules (26–28). To evaluate TML for QSAR learning we utilized 2,219 QSAR problems (24, 25). The baseline (intrinsic) QSAR representation was a 1,024-bit molecular fingerprint representation, which has previously been shown to be effective (25). For each ML method (RF, SVM, k-NN, and NN) we generated the TML extrinsic attributes by predicting compound activities using the previously learned ML models (see *SI Appendix*, *QSAR Learning*) then learned the TML QSAR models using the same ML method. Use of TML outperformed baseline ML for all methods. The results are given in Table 1. We found that the best overall results were for stacked TML XGB, which achieved a 7% overall improvement over baseline XGB, followed by TML NN. It is noteworthy that these datasets have been extensively studied [18 learning methods and 6 molecular representations (25)], and TML significantly outperformed the best previous results.

For our second problem domain we used the Library of Integrated Network-based Cellular Signatures data (LINCS) (29), which describes the measured expression levels of 978 landmark human genes under 118,050 experimental conditions. We cast the ML task as learning a model for each gene able to predict its expression level given experimental conditions (cell type, drug, and dosage) (see *SI Appendix*, *Gene Expression Learning*). The problem domain is suitable for TML as there are relationships between genes (homologies, common signaling pathways, etc.) and between experimental conditions (drug similarity, etc.) that can be exploited to improve performance. Using the same methodology as on the QSAR problem we performed a comparative assessment of RF, SVMs, k-NN, and NNs on the original intrinsic representation and the TML representation. The results for the problem are given in Table 1. Use of TML outperformed baseline ML for all methods. We found that the best overall result was for TML RF with a 4% overall improvement over baseline RF, followed by TML XGB and TML SVM.

Our third evaluation problem area was from ML, where a fundamental problem is to select the best ML method to use on a new learning task. An effective approach to this task is to use ML to solve this problem, which is a kind of metalearning (30). The ML task is to learn a metamodel to predict the performance of an ML method (given an exact configuration) on a new task, given the characteristics of the training data (e.g., statistics of the training data distribution). The problem domain is suitable for TML as ML tasks can be related by having similar data distributions and data properties (e.g., missing values) or by containing data being generated by similar processes. From OpenML (31) we took a set of 10,840 evaluations on 351 tasks (datasets) and 53 ML methods, which produced 351 learning tasks (*SI Appendix*, *Meta-Learning for Machine Learning*). The results for the problem are given in Table 1. Use of TML outperformed baseline ML for all methods. We found that the best overall result was TML stacking RF, with a 50% improvement over RF. A similar level of improvement was found for TML XGB over baseline XGB, with TML SVM and TML NN producing the best SVM and NN results. For k-NN stacking TML performed best. The percentage improvement with TML was much greater with the other tasks. This may be due to the fact that the original (intrinsic) features are rather weak descriptors of the training datasets, while the TML features encode much more implicit information about how algorithms behave on different tasks. In addition, measuring predictive performance has lower empirical noise than the other problem areas.

An increasingly important branch of ML is explainable AI, for in many applications (e.g., medical or financial) there is a necessity to make predictions understandable (32). In science, explainable ML predictions lead to new scientific insights. The understandability of ML models depends on model simplicity and on how closely the model reflects human concepts. The standard theory of the structure of concepts originated with Aristotle and is based on the presence of necessary and sufficient conditions that define and explain a concept. The explainability of TML models is based on the alternative approach of relating concepts based on the similarity between prototypes (33, 34).

Using RF models, and the domain of drug design, we illustrate the use of TML models to generate scientific understanding in three ways. In the first we illustrate how TML models can be used to provide insight into QSAR predictions for the specific drug target *H. sapiens* DHFR. Table 2 shows the top 10 attributes (baseline models) found to be important for predicting *H. sapiens* DHFR drug activity. As expected, there are other DHFR-targeted models in the list of important attributes, but intriguingly these are bacterial (*Lacticaseibacillus casei*, *Escherichia coli*, and *Mycobacterium avium*) and not mammalian. These three bacterial DHFR models triangulate the predictions for human DHFR: *L. casei* DHFR has the highest weight, suggesting it is most human-like, while *E. coli* and *M. avium* DHFRs differ significantly as *E. coli* DHFR strongly binds the antibiotic trimethoprim, while *M. avium* DHFR is resistant. This information can be used operationally to help design better human DHFR inhibitors to treat cancer. The other attributes (baseline models) in Table 2 provide similar insight.

**Table 2.** Top 10 models used to predict *H. sapiens* DHFR activity

Order	Target ID	Weight	Name	Species
1	CHEMBL2902	13.5	Dihydrofolate reductase	*L. casei*
2	CHEMBL5372	12.2	Methionyl-tRNA synthetase	*Staphylococcus aureus*
3	CHEMBL3048	10.6	Nitric-oxide synthase, brain	*Rattus norvegicus*
4	CHEMBL2111414	8.6	Tyrosine-protein kinase ABL	*H. sapiens*
5	CHEMBL329	8.5	Type-1A angiotensin II receptor	*R. norvegicus*
6	CHEMBL2014	7.4	Nociceptin receptor	*H. sapiens*
7	CHEMBL5491	7.2	Serine/threonine-protein kinase WEE1	*H. sapiens*
8	CHEMBL5441	7.2	Dihydrofolate reductase	*E. coli*
9	CHEMBL5457	6.8	Dihydrofolate reductase	*M. avium*
10	CHEMBL1075294	6.6	Indoleamine 2,3-dioxygenase 1	*M. musculus*

The seven non-DHFR targets seem to have little in common with DHFR; however, digging deeper provides biological insight. It is interesting that *H. sapiens* Tyrosine-protein kinase ABL was selected as it has been empirically shown that it is possible to jointly target *H. sapiens* DHFR and tyrosine kinases (42). The use of Nitric-oxide synthase (NOS), and the Serine/threonine-protein kinase WEE1, is also interesting. All known DHFR inhibitors share a bifurcated H-bond of an “aminopyridine” unit to glu30 in DHFR. In NOS there is a glutamate residue that binds the arginine over the heme with a similar bifurcated H-bond, and many NOS inhibitors mimic the arginine by being amidines, or amino pyridines etc. It is therefore possible that the selection of NOS is picking out the shared requirement for a donor/acceptor interaction to glutamic acid. It is also noteworthy that all kinase inhibitors hinge binders also share a similar donor acceptor interaction, which might also explain the kinase model.

TML can also be used to provide scientific insight through clustering (unsupervised learning). In chemoinformatics a fundamental problem is the estimation of the similarity of chemical compounds. The standard approach is to base similarity on similarity of chemical structure; commonly used methods are Tanimoto (Jaccard) coefficient distance between molecular fingerprints and graph similarity. However, what is generally of interest when comparing drugs is not structural similarity but functional similarity (15). This functional similarity can be measured using the accumulated information from empirical experiments encoded in QSAR models to calculate drug profiles: their predicted activity on drug targets (Fig. 2*A*). Such profiles can then be used to estimate distances between drugs and understand their pharmacology (see *SI Appendix*, *Clustering*). Fig. 2*B* shows a section of the TML-based clustering of Food and Drug Administration (FDA)-approved drugs with three clusters and associated compounds. Although the pharmacology of these compounds is complex, it is known to be based on a nexus of serotonin and dopamine receptor interactions. These interactions are correctly predicted by TML models and used in the clustering (see *SI Appendix*, *Predicted Drug Activities*). The pharmacology of compounds is explained by their relative position in the clustering (Fig. 2*B*).

We applied an analogous approach to the bioinformatic problem of estimating the similarity of protein targets (Fig. 2*C*) (see *SI Appendix*, *Clustering*). The standard approach to this task is to use evolutionary distance estimated by sequence comparison. However, what is most important in most problems is not evolutionary distance but the functional similarity of protein active sites. We can estimate this using the accumulated information in the TML QSAR models. For each of the targets we calculated its drug-activity profile: the predicted activity of FDA-approved compounds on the target. As with chemical compounds we argue that this clustering is more informative in drug design than conventional evolutionary distance, as it is based on how the target empirically responds to chemical compounds. One intriguing cluster of proteins (drug targets) identified by the similarity of their QSAR predictive models is shown in Fig. 2*C*. Although the proteins in the cluster do not share any obvious structural similarity, there is a clear theme to the function of these (mammalian) proteins, related to control of metabolism.

It is instructive to compare TML with the currently most significant form of ML, that of deep neural networks (DNNs) (35). DNN input is typically spatially or sequentially structured, and prior knowledge of structure is encoded in the structure of the network. This learned structure is latent. The success of DNNs is based on their ability to utilize multiple neural network layers, and very large amounts of data, to learn how to map poor input representations (e.g., image pixel values) into rich and effective latent representations. This is achieved through use of a differentiable learning model and end-to-end learning. The ability to improve weak input representations has enabled DNNs to succeed in domains that had previously proved recalcitrant to ML: beating World Champions at games such as Go (36), diagnosing skin cancers better than human specialists (9), and so on. A key lesson from the success of DNNs is therefore to use ML to enhance ML representations—which is precisely what TML does. DNNs are most applicable to problems where there are large amounts of available data to learn good representations and where there is no essential requirement for human-friendly symbolic models. These criteria are not met in most scientific problem areas.

The standard DNN approach to multitask problems is to learn a single large model that encompasses all the problems. In this approach, and in contrast to TML, neither the relationships between problems nor the relationships between examples are made extrinsic in the form of transformed features. For multitask problems, TML also has the advantage of enabling incremental learning: If new data or a new task is added, then each task model need not necessarily immediately be relearned. TML adds some additional computational costs to learning, but the additional cost of TML is low compared to DNN learning.

**Fig. 3.**
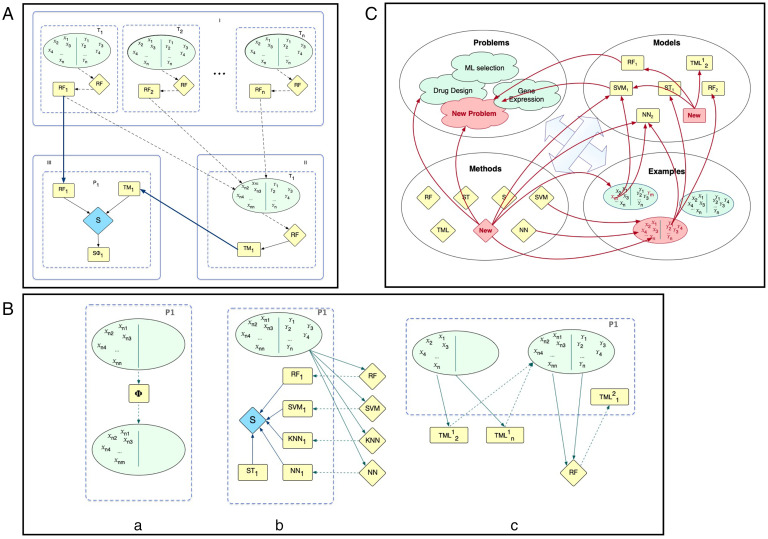
(*A*) Baseline TML. Each learning task is illustrated as an oval, models are squares, and learning methods diamonds (for clarity we only show RF). We focus on learning task T_1_. In (i) baseline learning is used to learn RF models for all the tasks (problems). In (ii) the predictions from the different learned RF models are used to learn a TML model for task T_1_. In (iii) stacking is used to combine the RF model RF_1_ and TML model TM_1_ to form the output stacking model S*Φ.* (*B*) Variants of TML. The figure illustrates three possible variants of TML applied to the same problem P1. (I) Shows TML feature selection. This selection could be done using baseline ML feature selection methods, or based on understanding the semantics of the relationships between the tasks (e.g., in drug design one might wish to consider the homology of related targets), and so on. (II) Illustrates the use of ensemble learning at the TML level. RF, SVM, k-NN, and NN are combined together using stacking. (III) Illustrates second-order (multilevel) TML. (*C*) ML as an ecosystem. Currently ML tasks are generally seen as to be solved stand-alone, or perhaps in small groups by MTL and TL. The TML-inspired view is that learning is cumulative and never-ending: When learning a new task, one should utilize existing models (knowledge) even if they were learned for different (but related) tasks, and when new problems, methods, models, and examples appear these should be used to improve existing models and predictions.

We have presented only the most basic form of TML (Fig. 3*A*). Many other variants with potentially greater performance are possible. For example, it is possible to integrate TML with feature selection and stacking in multiple possible ways (Fig. 3*B*). Given that TML can often produce better predictions than the original intrinsic representation, it is natural to extend the idea of TML by applying it a second time, i.e., to use the predictions from the transformed representation to form a second-order transformed representation (Fig. 3*B*). It is of course also possible to combine feature selection, ensemble learning, stacking, TML, second-order TML, and so on.

The traditional approach to ML is to view each learning task as a separate problem. This view is starting to change with progress in MTL (10), TL (13), life-long learning (37), and so forth. TML leads to an even broader vision of ML as an ecosystem (Fig. 3*C*). In this ecosystem, learning tasks, learning examples, ML methods, ML predictions, meta-ML methods, and so on all interact synergistically to produce enhanced performance and understanding over all tasks in the ecosystem. If more training examples are added, then both the specific task model is improved (using feature selection, ensemble learning, stacking, TML, second-order TML, etc.) and all the other models that use the task model (TML, second-order TML, etc.). Similarly, if a new task is added, then the new task model is used to extend the transformed representation, and hence improve all the other task models improved through TML, second-order TML, and so forth. If a new ML or meta-ML method is added, then all the task models are incrementally improved (Fig. 3*C*). In such an ML ecosystem, as new knowledge is added predictive performance will incrementally improve (38). The predictions will also be more robust, as prior knowledge from many different sources is used in any prediction (38).

Within ML there is increasing interest in the automation of ML, and there exist a number of both free and commercial systems that automate the application of ML to new problems (39). For example, Auto-WEKA and Auto-sklearn (39) search through a space of possible ML methods, and hyper-parameters, to optimize ML predictions. However, no current ML automation system has discovered a valuable new ML idea such as dropout, stacking, and so on. Although there is increasing amount of research on AI systems designed to automate scientific discovery (40), and these systems are heavily based on ML, little work has been done on applying AI discovery systems to ML. The development of a ML system able to learn important new ML ideas would transform ML and the world.

## Materials and Methods

To enable reproducibility, all of the thousands of datasets (QSAR, LINCS, and Metalearning), the links to the code (TML, RF, XGB, SVM, k-NN, NN), and the ∼50,000 ML RF (counting all decision trees) models are available under the creative commons license at the Open Science Platform: https://osf.io/vbn5u/. This amounts to ∼100 Gbytes of compressed data. Few ML projects have put online so much reusable data. To maximize its added value we follow the FAIR (Findability, Accessibility, Interoperability, and Reusability) principles for publishing digital objects (41) (see *SI Appendix*, *FAIR Sharing*).

## Supplementary Material

Supplementary File

## Data Availability

Datasets, code, and ML models reported in this study have been deposited in Open Science Framework (https://osf.io/vbn5u/).
