# Supplementary material for: Transformational machine learning: Learning how to learn from many related scientific problems
Source: Proc Natl Acad Sci U S A. 2021 Nov 29;118(49):e2108013118. doi: 10.1073/pnas.2108013118 (PMC8670494; doi:10.1073/pnas.2108013118)
Supplement: Supplementary File [file pnas.2108013118.sapp.pdf]

Supporting Information

QSAR Learning

We extracted 2,219 protein targets from ChEMBL with a diverse number of drug-like chemical compounds, ranging from 30 to about 6,000, each target resulting in a dataset with as many examples as compounds. Although we could have used a larger set of targets, we limited the analysis to the ones previously used in (Olier *et al*., 2018). The data is available from the Mendeley Data Repository at http://dx.doi.org/10.17632/spwgrcnjdg.1. Chemical compounds were intrinsically described using a standard fingerprint representation (as it is the most commonly used in QSAR learning), where the presence or absence of a particular molecular substructure in a molecule (e.g. methyl group, benzene ring) is indicated by a Boolean variable. Specifically, we used the RDKit to calculate the 1024 bits FCFP4 fingerprint representation, which is one of the extended-connectivity fingerprints (Rogers and Hahn, 2010) for molecular characterisation. In Olier *et al*., 2018, it was shown that FCFP4-based datasets exhibited best overall performance over several other fingerprints of commonly used. Each dataset consisted of 1,024 input binary variables, one for each fingerprint bit, and one floating-point output variable which represented the chemical compound activities against the target. We used IC50 values, inhibitory drug concentrations at 50%. IC50 value states the concentration of the drug compound that is required to block or inhibit 50% of the proteins. This response data has been normalised by taking the negative log of the drug concentrations that inhibited 50% of a target (pXC50):

$$pXC50 = -log_{10}IC50$$

The pXC50 provides a continuous scale of 1–12 where a compound of the value 1 is the least potent inhibitor and requires a large concentration of the drug to achieve 50% inhibition and 12 is the most potent inhibitor requiring a very low concentration to achieve 50% inhibition. In a small proportion of cases, where multiple activities have been reported for a particular compound-target pair, a consensus value was selected as the median of those activities falling in the modal log unit. Therefore, the unit of activity we are referring to is the pseudo-pIC50. In the end, we understood the problem of learning QSARs as the regression task of predicting the pXC50 activity given a chemical compound represented with the 1,024-FCFP4 fingerprint. TML dataset input columns are formed using the predicted pXC50 activities from the baseline models on the test subsets of each CV iteration. TML dataset output columns are the same pXC50 activities as used for the baseline datasets.

We employed five machine learning algorithms that are deemed very popular and their implementations, easily accessible from R: random forest (RF, as implemented in the *range* R package), support vector machine (SVM, *ksvm* R package), k-nearest neighbour (KNN, *FNN* R package), neural networks (NN, *tensorflow.keras* python package), and extreme gradient boosted trees (XGB, XGBoost R package). Hyperparameters were selected as follows: in all RF experiments, we used 500 trees, a third of the total number of variables were considered at each split, and five observations were used in each terminal node. For the experiments with SVMs, we used RBF kernels with gamma value of 0.5 and cost of 1.0. The chosen RF and SVM hyperparameter sets were the ones that produced best overall performance after having been tested on a smaller subset of datasets randomly selected. For KNN, the number of neighbours ('k') was chosen individually for each QSAR model using an inner cycle of 3-fold cross-validation. For NNs, we tested on a small subset of the datasets several fully connected feedforward architectures. In addition, we used dropping-out and L2-penalisation at different rates in order to minimise the risk of overfitting. In the end, we chose for the baseline experiments an architecture that consisted of 1 hidden layer with 128 neurons and 1 output neuron. ReLU activation functions were used in the hidden layer, whilst the output neuron had a linear

function as traditionally used in regression problems. For the TML experiments, the NN architecture consisted of 2 hidden layers, the first one with 712 neurons and the second one, with 128, and both with ReLUs. We used *ADAM* as the optimiser in both set of experiments. For the baseline and TML experiments, XGB's hyperparameters for each dataset model were chosen by exploring the following grid: number of rounds values 1000 and 1500, learning rate values in 0.001, 0.01, 0.1, 0.2, and 0.3. The hyperparameter set producing the best model performance was chosen using an inner validation split of 30%.

The stacking experiments were performed using convex linear regression and ridge regression. The linear regression model was set up such that the weights of the coefficients are non-negative and sum to 1. The ridge regularization parameter was tuned using internal cross-validation. We assessed the performance of all the models using 10-fold cross-validation. The code is available at https://github.com/iaolier/TML-QSAR.

Gene Expression Learning

We utilized the Library of Integrated Network-based Cellular Signatures data (LINCS). This data describes the effect of drugs in cancer cell lines on the expression levels of 978 landmark human genes. We used LINCS Phase II data (accession code GSE70138), which consists of 118,050 experimental conditions, along with the corresponding expression levels for 978 landmark genes. We generated attributes for each perturbation condition using the accompanying metadata. Each experimental condition is associated with a perturbagen (drug), cell type and site, perturbagen dosage, and perturbagen time frame. In total, there are 30 cell types (ct), 14 cell sites (cs), 83 dosages (d) and 3 time points (tp). Of the 2,170 drugs in the dataset, 1,795 have valid chemical structures (canonical smiles codes) according to the metadata. We converted the canonical SMILES to the a 1,024 bit FCFP4 finger-prints (fp) using RDKit (Landrum, 2016). For all perturbation conditions with valid canonical smiles as rows, we generated Boolean features with the following columns: [ct1 . . . ct30][cs1 . . . cs14][d1 . . . d83][tp1 . . . tp3][fp1 . . . fp1024]. This generated a 107,152 by 1,155 experimental condition matrix, row and column identifiers included, which can be used as input for building models to predict the expression levels of the 978 genes using traditional machine learning techniques. For each gene we generated both a train and test set with 7,000 and 3,000 samples respectively. We did this by first randomly splitting the original perturbation condition data with 107,152 samples and their corresponding gene expression levels, into train and test sets of 70% and 30% respectively. Using this main train and test set, we randomly sampled train and test individuals for each gene. The gene expression levels for the 978 genes were normalised such that their values lie between 0.0 and 1.0.

We used five learning algorithms: random forests (RF), gradient boosting machines (XGBoost), support vector machines (SVM), k-nearest neighbors (KNN), and neural networks (NN). For RF 500 trees were grown, a third of the total number of variables were considered at each split, and five observations were used in each terminal node. For XGBoost, most hyperparameters were left in their default setting while the following were tuned using a grid: number of rounds = (500, 1000, 1500, 2000), max depth = (2, 4, 6, 8), and learning rate = (0.001, 0.01, 0.1, 0.2, 0.3). The SVMs were built using an epsilon of 0.01, cost of 0.25, and gamma of 0.5, having learned that these values perform reasonably well through data exploration. Five neighbors were used for KNN. We used a NN with two hidden layers, the first layer contains a third of the total number of input nodes, and the second layer contains a third of the number of nodes in the first layer. Since it is a regression problem, there was only one output node. All experiments were performed in R and the code and dataset are available at https://github.com/oghenejokpeme/TML-gene-expression and http://dx.doi.org/10.17632/2djzy3p9p9.1 respectively.

Due to computational expense, we performed the transformative experiments using a sequential monotonic increase of the number of input features. That is, having performed the baseline case experiments for a given learner, we do not then perform the transformative case for a gene using 977 gene features. Instead, we perform the transformative case using 50 features, then 100 and so on, such that all the gene features in the previous set are also present in the current set. The results from the transformative models reported in the main manuscript were built using 500 features for RF and SVM, 300 for KNN, and 50 for NN. The stacking experiments were performed using convex linear regression and ridge regression. The linear regression model was set up such that the weights of the coefficients are non-negative and sum to 1. The ridge regularization parameter was tuned using internal cross-validation.

Meta-Learning for Machine Learning

The third problem domain is in meta-learning for machine learning. The specific problem consisted in predicting the performance of a machine learning method (given an exact configuration) on a new task, given the characteristics of the training data (e.g. statistics of the training data distribution). Domain problems are assumed to be related by having similar data distributions, data defects (e.g. missing values), or by containing data being generated by similar processes. The properties used to describe the datasets themselves are typically called meta-features.

From OpenML we retrieved data from an earlier meta-learning study (Details can be found on https://www.openml.org/s/7). Although we had to exclude a few tasks and algorithms because they lacked sufficient evaluations in OpenML, this yielded a set of 10840 evaluations on 351 tasks (datasets) and 53 machine learning methods (called flows on OpenML) from mlr (Bischl et al., 2016). From each task, 21 dataset descriptors were extracted, such as the number of examples, number of missing values, and percentage of numeric features. We formed meta-datasets, one for each machine learning method. An observation within a meta-dataset represents an original OpenML task, and each feature, a dataset descriptor. The original aim of the study was to predict the area under the ROC (AUC). Therefore, in total, we produced 53 meta-datasets with a diverse number of OpenML tasks, ranging from above 100 to about 250. We applied transformative learning to transform the original representation of the datasets into extrinsic descriptors of the OpenML tasks. Similarly to the other two problem domains, five ML algorithms were selected to do the transformation: RF (500 trees), SVM (RBF kernel, gamma = 0.5, C=1.0), KNN (‘k’ chosen by an internal 3-fold cross-validation cycle), and NN (1 hidden layer, 10 hidden neurons with ReLU activation functions, 1 output neuron with linear activation function), and XGB (similar to the other two problems). The transformed descriptors were generated by predicting AUC using all available models - excluding the one from the which the OpenML task belonged. In this way 52 extrinsic descriptors were generated for each OpenML task. Model performances were assessed using 10-fold cross-validation. The baseline models were manually tuned. For the RF models, the main hyperparameters are the number of trees and the number of random features used in each split (mtry). After several rounds of experimentation, we used 500 trees for every model, and used the default heuristic (the square root of the number of features) to set the mtry hyperparameter depending on the dataset. Further tuning did not significantly improve performance.

The data used for these experiments is available from Mendeley Data Repository at http://dx.doi.org/10.17632/7xx7ty87x2.1 and the code is available at https://github.com/iaolier/TML-metalrn.

Clustering chemical compounds and protein targets

To learn whether the TML descriptions of the chemical compounds express valuable relationships between them, we clustered them using hierarchical clustering with the HDBSCAN algorithm [McInnes2017]. To make the clustering pharmacologically relevant we focussed on the highest activities, and only kept the 10% highest activities and set the remainder to 0. This results in a sparse representation. Columns that become entirely 0 are removed for the clustering. These correspond to the protein targets for which no high activities are predicted for any drug. Because of the high dimensionality of this data, we used a normalized Euclidean distance to obtain the hierarchical clusterings. To select flat clusters from the cluster tree hierarchy, we used the Excess of Mass selection method, and a minimal cluster size of 3.

To cluster the protein targets, we follow exactly the same procedure, but now on the transpose of the sparse matrix. Since this matrix has drug compounds in the rows and protein targets in the columns, the transpose yields a representation of the protein targets in terms of the predicted activities for all known drugs. Again, we only keep the 10% highest activities and remove the columns that become entirely zero (the chemical compounds that don't have a high activity on any protein target). The hierarchical clustering was again done with the HDBSCAN algorithm and normalized Euclidean distances.

[McInnes2017]: McInnes et al, (2017), hdbscan: Hierarchical density based clustering, Journal of Open Source Software, 2(11), 205, doi:10.21105/joss.00205

To estimate distances between the chemical compounds, and produce figures 3b and 3c, we performed dimensionality reduction using the tSNE algorithm [vanderMaaten2008]. In Figures 3a and 3b, we embedded the data in two dimensions, and color-coded the chemical compounds and protein targets according to their assigned clusters (learned with HDBSCAN as described above), using black for the 'singleton' elements that do not belong to any cluster. The code to produce the clustering can be found on: https://github.com/joaquinvanschoren/transformational-learning

[vanderMaaten2008]: van der Maaten L. and Hinton G. (2008), Visualizing Data using t-SNE, Journal of Machine Learning Research 9(86), 2579-2605

FAIR Sharing

To enable reproducibility, all of the thousands of datasets (QSAR, LINCS, and Meta-learning), the links to the code (TML, RF, XGB, SVM, KNN, NN), and the ~50,000 ML random forest models (counting all decision trees) models are available under the creative commons license at the Open Science Platform: https://osf.io/vbn5u/ This amounts to ~100GBs of compressed data. Few ML projects have put online so much reusable data.

To maximize its added-value, we follow the FAIR (Findability, Accessibility, Interoperability, and Reusability) principles for publishing digital objects (Wilkinson *et al*., 2016). The aim of FAIR movement is to produce machine-actionable digital resources, which will facilitate discovery, evaluation, data and knowledge integration and reuse by the community.

We employed MLS (Machine Learning Schema) as a set of metadata to describe the reported prediction models (https://github.com/ML-Schema). MLS is specifically designed to capture the most essential information about machine learning studies. It also provides mapping to others machine learning vocabularies and ontologies (i.e. DMOP (Keet *et al*. 2015), Expose (Vanschoren & Soldatova, 2010), OntoDM (Panov *et al.*, 2014) and MEX (Esteves *et al*., 2015)). MLS enabled us to capture such metadata about the reported models as input (e.g. LINCS), algorithm (e.g. Random Forest Regression), and model evaluation (e.g. root Mean Square Error). Additionally, we employed other formalisms:

·DC (Dublin Core) (https://www.dublincore.org) terms to capture provenance information (i.e. authors or creators) and information about license (i.e. CC BA-SY 2.0) (https://creativecommons.org/licenses/by-sa/2.0/).

· IAO (Information Artifact Ontology) (http://www.obofoundry.org/ontology/iao.html) terms to capture information about programming languages, models, and their versions.

· BIOPAX (Biological Pathway Exchange) ontology (www.biopax.org) to capture information about genes in the datasets and models.

The whole set of the metadata used for our transformative learning study is encoded as a tml-metadata ontology. It is available at: www.purl.org/TML and https://bioportal.bioontology.org/ontologies/TML in the following formats: OWL, CSV, RDF, XML.

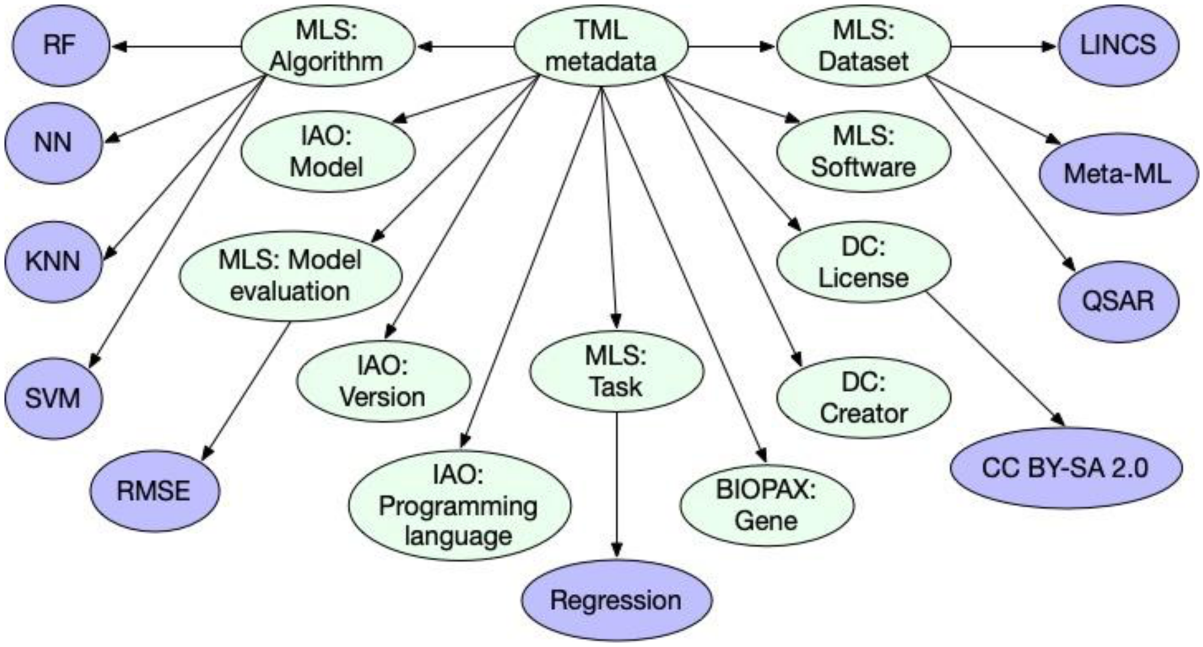

Fig 1: TML metadata terms are depicted in green and example instances are in purple, The relationships between the terms are is-a, and between the terms and instances are instance-of.

More specifically, tml-metada set includes eleven terms:

1. MLS: Algorithm (regardless software implementation)
2. DC: Creator (an entity primarily responsible for making the resource)

3. BIOPAX: Gene (a part of a nucleic acid that contains all the necessary elements to encode a functional transcript)
4. DC: License, i.e. CC BY-SA 2.0 https://creativecommons.org/licenses/by-sa/2.0/
5. MLS: Dataset (LINC dataset, Meta-ML dataset, QSAR dataset)
6. IAO: Model (a generalization of a set of training data able to predict values for unseen instances. It is an output from an execution of a data mining algorithm implementation)
7. MLS: Model evaluation (a setting of a value of the performance measure specified by the evaluation specification. It connects a measure specification with its value)
8. IAO: Programming language (a language in which source code is written, intended to executed/run by a software interpreter. Programming languages are ways to write instructions that specify what to do, and sometimes, how to do it).
9. MLS: Software (is implemented computer programs, procedures, scripts or rules with associated documentation, possibly constituting an organized environment, stored in read/write memory for the purpose of being executed within a computer system)
10. MLS: Task (a formal description of a process that needs to be completed (e.g. based on inputs and outputs). A Task is any piece of work that needs to be addressed in the data mining process. In ML Schema, it is defined based on data)
11. IAO: Version (an information content entity which is a sequence of characters borne by part of each of a class of manufactured products or its packaging and indicates its order within a set of other products having the same name).

For example, one of the thousands of produced in this study models is the AARS-Random Forests predictive model. Its metadata capture the following information about the model and the process of its generation: The input data is the LINCS dataset, the employed algorithm is Random Forests, the model was evaluated using RMSE, the model includes such gene as AARS and available under the CC BY-SA 2.0 license.

Table 1 provides a summary of how the recording and publishing of tml study outputs are compliant with each of FAIR principles.

Table 1: Compliance with FAIR principles.

**Findable**		
F1: metadata are assigned a globally unique and persistent identifier	tml identifiers are mapped to corresponding PURLs provided by DC and IAO where appropriate, e.g. the term IAO: version (http://purl.obolibrary.org/obo/IAO_0000129)<br>tml identifiers are registered at www.purl.org/TML	√

F2: data are described with rich metadata	We used a combination of MLS (Machine Learning Schema), DC (Dublin Core) and IAO (Information Artefact Ontology) terms as metadata	√
F3: metadata clearly and explicitly include the identifier of the data it describes	Each predictive model has an identifier (the name of each model is unique and informative); model metadata clearly and explicitly identify each model as an instance of the class IAO: model	√
F4: metadata are registered or indexed in a searchable resource	Tml is registered and searchable at www.purl.org/TML and https://bioportal.bioontology.org/ontologies/TML	√
**Accessible**		
A1: metadata are retrievable by their identifier using a standardized communications protocol	HTTP	√
A2: metadata are accessible, even when the data are no longer available	Tml metadata is accessible at www.purl.org/TML and https://bioportal.bioontology.org/ontologies/TML	√
**Interoperable**		
I1: metadata use a formal, accessible, shared, and broadly applicable language for knowledge representation.	RDF	√
I2: metadata use vocabularies that follow FAIR principles	Both IAO, MLS and DC are FAIR	√
I3: metadata include qualified references to other metadata	The original source of each term is explicitly attributed in tml	√

Reusable		
R1: data are richly described with a plurality of accurate and relevant attributes	Each predictive model is described by a set of attributes to capture information about how it was produced and evaluated, for what task, and using what inputs.	√
R1.1: data are released with a clear and accessible data usage license	CC BA-SY	√
R1.2: data are associated with detailed provenance	The class 'DC: creator' is used to list authors of the reported digital objects	√
R1.3: (meta)data meet domain-relevant community standards	There are several proposed standards for reporting ML studies. MLS is a mapping between major representations. MLS is a proposed W3C community standard.	√

References for the FAIR sharing section

1. Keeta C.M., Ławrynowicz A., d'Amatoc C., et al. (2015) The Data Mining OPtimization Ontology. J. of Web Semantics, v. 32, pp. 43-53.
2. Vanschoren, J., Soldatova, L. (2010). Exposé: An ontology for data mining experiments. In Proc. SoKD/ ECML pp. 31-46.
3. Panov, P., Soldatova, L.N., Dzeroski, S. (2014) Ontology of Core Data Mining Entities. J. of Data Mining and Knowledge Discovery. doi:10.1007/s10618-014-0363
4. Esteves D., Moussallem D, Neto C., et al (2015) MEX Vocabulary: A Lightweight Interchange Format for Machine Learning Experiments. In Proc. SEMANTiCS

Predicted Drug Activities

```
1. molecule_id,target_id,pred_act,label,altlabel,pref_name,organism,n_co
   mpounds
2. CHEMBL1185,CHEMBL1805,7.18147405,Zomig,Zolmitriptan,Serotonin 1f (5-
   HT1f) receptor,Homo sapiens,126
3. CHEMBL1185,CHEMBL1833,6.574624898,Zomig,Zolmitriptan,Serotonin 2b (5-
   HT2b) receptor,Homo sapiens,1238
4. CHEMBL1185,CHEMBL1983,8.492942184,Zomig,Zolmitriptan,Serotonin 1d (5-
   HT1d) receptor,Homo sapiens,1251
```

5. CHEMBL1185,CHEMBL214,7.04000968,Zomig,Zolmitriptan,Serotonin 1a (5-HT1a) receptor,Homo sapiens,3356
6. CHEMBL1185,CHEMBL228,7.052493037,Zomig,Zolmitriptan,Serotonin transporter,Homo sapiens,4156
7. CHEMBL1185,CHEMBL2490,7.227832236,Zomig,Zolmitriptan,Serotonin 2a (5-HT2a) receptor,Sus scrofa,99
8. CHEMBL1185,CHEMBL265,7.529636416,Zomig,Zolmitriptan,Dopamine D1 receptor,Rattus norvegicus,992
9. CHEMBL1185,CHEMBL2967,5.439738495,Zomig,Zolmitriptan,Dopamine D1 receptor,Bos taurus,285
10. CHEMBL1185,CHEMBL313,7.558857749,Zomig,Zolmitriptan,Serotonin transporter,Rattus norvegicus,3527
11. CHEMBL1185,CHEMBL3223,7.372549695,Zomig,Zolmitriptan,Serotonin 7 (5-HT7) receptor,Rattus norvegicus,365
12. CHEMBL1185,CHEMBL339,8.617444581,Zomig,Zolmitriptan,Dopamine D2 receptor,Rattus norvegicus,4378
13. CHEMBL1185,CHEMBL3426,5.254248717,Zomig,Zolmitriptan,Serotonin 5a (5-HT5a) receptor,Homo sapiens,466
14. CHEMBL1185,CHEMBL3427,6.805579914,Zomig,Zolmitriptan,Dopamine D2 receptor,Mus musculus,167
15. CHEMBL1185,CHEMBL3459,6.872172774,Zomig,Zolmitriptan,Serotonin 1b (5-HT1b) receptor,Rattus norvegicus,397
16. CHEMBL1185,CHEMBL3666,6.575011867,Zomig,Zolmitriptan,Serotonin 3a (5-HT3a) receptor,Cavia porcellus,51
17. CHEMBL1185,CHEMBL4105,7.604360636,Zomig,Zolmitriptan,Serotonin 1d (5-HT1d) receptor,Sus scrofa,127
18. CHEMBL1185,CHEMBL5377,4.436797173,Zomig,Zolmitriptan,Serotonin 2a (5-HT2a) receptor,Mus musculus,68
19. CHEMBL1185,CHEMBL5450,6.804122297,Zomig,Zolmitriptan,Serotonin 1d (5-HT1d) receptor,Rattus norvegicus,48
20. CHEMBL1201087,CHEMBL1805,7.189043934,Cabaser,Cabergoline,Serotonin 1f (5-HT1f) receptor,Homo sapiens,126
21. CHEMBL1201087,CHEMBL1898,7.134173075,Cabaser,Cabergoline,Serotonin 1b (5-HT1b) receptor,Homo sapiens,1236
22. CHEMBL1201087,CHEMBL1983,7.906976486,Cabaser,Cabergoline,Serotonin 1d (5-HT1d) receptor,Homo sapiens,1251
23. CHEMBL1201087,CHEMBL214,7.6318575,Cabaser,Cabergoline,Serotonin 1a (5-HT1a) receptor,Homo sapiens,3356
24. CHEMBL1201087,CHEMBL224,6.863965824,Cabaser,Cabergoline,Serotonin 2a (5-HT2a) receptor,Homo sapiens,2778
25. CHEMBL1201087,CHEMBL225,6.704141587,Cabaser,Cabergoline,Serotonin 2c (5-HT2c) receptor,Homo sapiens,2869
26. CHEMBL1201087,CHEMBL228,6.994093664,Cabaser,Cabergoline,Serotonin transporter,Homo sapiens,4156
27. CHEMBL1201087,CHEMBL234,7.651542245,Cabaser,Cabergoline,Dopamine D3 receptor,Homo sapiens,3133
28. CHEMBL1201087,CHEMBL2490,6.787208854,Cabaser,Cabergoline,Serotonin 2a (5-HT2a) receptor,Sus scrofa,99
29. CHEMBL1201087,CHEMBL313,7.66173261,Cabaser,Cabergoline,Serotonin transporter,Rattus norvegicus,3527
30. CHEMBL1201087,CHEMBL3155,7.353755453,Cabaser,Cabergoline,Serotonin 7 (5-HT7) receptor,Homo sapiens,1171
31. CHEMBL1201087,CHEMBL3223,7.483659714,Cabaser,Cabergoline,Serotonin 7 (5-HT7) receptor,Rattus norvegicus,365
32. CHEMBL1201087,CHEMBL324,6.796355236,Cabaser,Cabergoline,Serotonin 2c (5-HT2c) receptor,Rattus norvegicus,575
33. CHEMBL1201087,CHEMBL3371,7.280173955,Cabaser,Cabergoline,Serotonin 6 (5-HT6) receptor,Homo sapiens,2408

34. CHEMBL1201087,CHEMBL339,6.778836787,Cabaser,Cabergoline,Dopamine D2 receptor,Rattus norvegicus,4378
35. CHEMBL1201087,CHEMBL3426,5.523532264,Cabaser,Cabergoline,Serotonin 5a (5-HT5a) receptor,Homo sapiens,466
36. CHEMBL1201087,CHEMBL3427,6.829242678,Cabaser,Cabergoline,Dopamine D2 receptor,Mus musculus,167
37. CHEMBL1201087,CHEMBL3459,7.278369761,Cabaser,Cabergoline,Serotonin 1b (5-HT1b) receptor,Rattus norvegicus,397
38. CHEMBL1201087,CHEMBL3597,7.084112898,Cabaser,Cabergoline,Serotonin 5a (5-HT5a) receptor,Mus musculus,40
39. CHEMBL1201087,CHEMBL3666,6.618371201,Cabaser,Cabergoline,Serotonin 3a (5-HT3a) receptor,Cavia porcellus,51
40. CHEMBL1201087,CHEMBL3737,6.918434212,Cabaser,Cabergoline,Serotonin 1a (5-HT1a) receptor,Mus musculus,70
41. CHEMBL1201087,CHEMBL4105,7.479244023,Cabaser,Cabergoline,Serotonin 1d (5-HT1d) receptor,Sus scrofa,127
42. CHEMBL1201087,CHEMBL5017,6.775978608,Cabaser,Cabergoline,Serotonin 4 (5-HT4) receptor,Cavia porcellus,273
43. CHEMBL1278,CHEMBL1805,7.522748557,Amerge,Naratriptan,Serotonin 1f (5-HT1f) receptor,Homo sapiens,126
44. CHEMBL1278,CHEMBL1833,6.584602072,Amerge,Naratriptan,Serotonin 2b (5-HT2b) receptor,Homo sapiens,1238
45. CHEMBL1278,CHEMBL1850,6.103601355,Amerge,Naratriptan,Dopamine D5 receptor,Homo sapiens,274
46. CHEMBL1278,CHEMBL1983,8.156948693,Amerge,Naratriptan,Serotonin 1d (5-HT1d) receptor,Homo sapiens,1251
47. CHEMBL1278,CHEMBL214,7.171497793,Amerge,Naratriptan,Serotonin 1a (5-HT1a) receptor,Homo sapiens,3356
48. CHEMBL1278,CHEMBL228,7.425794146,Amerge,Naratriptan,Serotonin transporter,Homo sapiens,4156
49. CHEMBL1278,CHEMBL2490,7.15971035,Amerge,Naratriptan,Serotonin 2a (5-HT2a) receptor,Sus scrofa,99
50. CHEMBL1278,CHEMBL313,7.874848922,Amerge,Naratriptan,Serotonin transporter,Rattus norvegicus,3527
51. CHEMBL1278,CHEMBL3371,7.136461096,Amerge,Naratriptan,Serotonin 6 (5-HT6) receptor,Homo sapiens,2408
52. CHEMBL1278,CHEMBL3426,5.365957625,Amerge,Naratriptan,Serotonin 5a (5-HT5a) receptor,Homo sapiens,466
53. CHEMBL1278,CHEMBL3427,6.860652603,Amerge,Naratriptan,Dopamine D2 receptor,Mus musculus,167
54. CHEMBL1278,CHEMBL3446,6.125871221,Amerge,Naratriptan,Serotonin 2a (5-HT2a) receptor,Bos taurus,128
55. CHEMBL1278,CHEMBL3459,6.89167966,Amerge,Naratriptan,Serotonin 1b (5-HT1b) receptor,Rattus norvegicus,397
56. CHEMBL1278,CHEMBL3597,6.600589161,Amerge,Naratriptan,Serotonin 5a (5-HT5a) receptor,Mus musculus,40
57. CHEMBL1278,CHEMBL3666,6.57761484,Amerge,Naratriptan,Serotonin 3a (5-HT3a) receptor,Cavia porcellus,51
58. CHEMBL1278,CHEMBL4105,7.477406883,Amerge,Naratriptan,Serotonin 1d (5-HT1d) receptor,Sus scrofa,127
59. CHEMBL1278,CHEMBL5453,6.094525066,Amerge,Naratriptan,Serotonin transporter,Macaca mulatta,67
60. CHEMBL128,CHEMBL1805,7.392229295,Imitrex,Sumatriptan,Serotonin 1f (5-HT1f) receptor,Homo sapiens,126
61. CHEMBL128,CHEMBL1833,6.667140734,Imitrex,Sumatriptan,Serotonin 2b (5-HT2b) receptor,Homo sapiens,1238
62. CHEMBL128,CHEMBL1850,5.894668136,Imitrex,Sumatriptan,Dopamine D5 receptor,Homo sapiens,274

63. CHEMBL128,CHEMBL1898,7.597804878,Imitrex,Sumatriptan,Serotonin 1b (5-HT1b) receptor,Homo sapiens,1236
64. CHEMBL128,CHEMBL1983,8.265591179,Imitrex,Sumatriptan,Serotonin 1d (5-HT1d) receptor,Homo sapiens,1251
65. CHEMBL128,CHEMBL214,6.879500215,Imitrex,Sumatriptan,Serotonin 1a (5-HT1a) receptor,Homo sapiens,3356
66. CHEMBL128,CHEMBL228,7.207583525,Imitrex,Sumatriptan,Serotonin transporter,Homo sapiens,4156
67. CHEMBL128,CHEMBL2490,7.323777206,Imitrex,Sumatriptan,Serotonin 2a (5-HT2a) receptor,Sus scrofa,99
68. CHEMBL128,CHEMBL313,7.672640689,Imitrex,Sumatriptan,Serotonin transporter,Rattus norvegicus,3527
69. CHEMBL128,CHEMBL3426,5.170642645,Imitrex,Sumatriptan,Serotonin 5a (5-HT5a) receptor,Homo sapiens,466
70. CHEMBL128,CHEMBL3427,6.928989453,Imitrex,Sumatriptan,Dopamine D2 receptor,Mus musculus,167
71. CHEMBL128,CHEMBL3446,6.13986167,Imitrex,Sumatriptan,Serotonin 2a (5-HT2a) receptor,Bos taurus,128
72. CHEMBL128,CHEMBL3459,7.136083684,Imitrex,Sumatriptan,Serotonin 1b (5-HT1b) receptor,Rattus norvegicus,397
73. CHEMBL128,CHEMBL3666,6.540578555,Imitrex,Sumatriptan,Serotonin 3a (5-HT3a) receptor,Cavia porcellus,51
74. CHEMBL128,CHEMBL4105,7.772667812,Imitrex,Sumatriptan,Serotonin 1d (5-HT1d) receptor,Sus scrofa,127
75. CHEMBL128,CHEMBL5377,4.439672538,Imitrex,Sumatriptan,Serotonin 2a (5-HT2a) receptor,Mus musculus,68
76. CHEMBL128,CHEMBL5450,6.874906548,Imitrex,Sumatriptan,Serotonin 1d (5-HT1d) receptor,Rattus norvegicus,48
77. CHEMBL1510,CHEMBL1805,7.043260173,Relpax,Eletriptan,Serotonin 1f (5-HT1f) receptor,Homo sapiens,126
78. CHEMBL1510,CHEMBL1833,6.816564629,Relpax,Eletriptan,Serotonin 2b (5-HT2b) receptor,Homo sapiens,1238
79. CHEMBL1510,CHEMBL1850,5.913493206,Relpax,Eletriptan,Dopamine D5 receptor,Homo sapiens,274
80. CHEMBL1510,CHEMBL1898,7.585627699,Relpax,Eletriptan,Serotonin 1b (5-HT1b) receptor,Homo sapiens,1236
81. CHEMBL1510,CHEMBL214,7.254489759,Relpax,Eletriptan,Serotonin 1a (5-HT1a) receptor,Homo sapiens,3356
82. CHEMBL1510,CHEMBL228,7.166275362,Relpax,Eletriptan,Serotonin transporter,Homo sapiens,4156
83. CHEMBL1510,CHEMBL234,7.131540535,Relpax,Eletriptan,Dopamine D3 receptor,Homo sapiens,3133
84. CHEMBL1510,CHEMBL2490,6.863460758,Relpax,Eletriptan,Serotonin 2a (5-HT2a) receptor,Sus scrofa,99
85. CHEMBL1510,CHEMBL313,7.805396784,Relpax,Eletriptan,Serotonin transporter,Rattus norvegicus,3527
86. CHEMBL1510,CHEMBL3155,7.315951327,Relpax,Eletriptan,Serotonin 7 (5-HT7) receptor,Homo sapiens,1171
87. CHEMBL1510,CHEMBL3223,7.3237706,Relpax,Eletriptan,Serotonin 7 (5-HT7) receptor,Rattus norvegicus,365
88. CHEMBL1510,CHEMBL3371,7.294217078,Relpax,Eletriptan,Serotonin 6 (5-HT6) receptor,Homo sapiens,2408
89. CHEMBL1510,CHEMBL339,6.799688615,Relpax,Eletriptan,Dopamine D2 receptor,Rattus norvegicus,4378
90. CHEMBL1510,CHEMBL3426,5.597625051,Relpax,Eletriptan,Serotonin 5a (5-HT5a) receptor,Homo sapiens,466
91. CHEMBL1510,CHEMBL3427,6.588791851,Relpax,Eletriptan,Dopamine D2 receptor,Mus musculus,167

92. CHEMBL1510,CHEMBL3459,6.519485751,Relpax,Eletriptan,Serotonin 1b (5-HT1b) receptor,Rattus norvegicus,397
93. CHEMBL1510,CHEMBL3597,6.73828936,Relpax,Eletriptan,Serotonin 5a (5-HT5a) receptor,Mus musculus,40
94. CHEMBL1510,CHEMBL3666,6.68877628,Relpax,Eletriptan,Serotonin 3a (5-HT3a) receptor,Cavia porcellus,51
95. CHEMBL1510,CHEMBL3737,7.275408498,Relpax,Eletriptan,Serotonin 1a (5-HT1a) receptor,Mus musculus,70
96. CHEMBL1510,CHEMBL4105,7.59382147,Relpax,Eletriptan,Serotonin 1d (5-HT1d) receptor,Sus scrofa,127
97. CHEMBL1510,CHEMBL5453,6.077053873,Relpax,Eletriptan,Serotonin transporter,Macaca mulatta,67
98. CHEMBL157138,CHEMBL1805,7.172643203,Lysuride,Lisuride,Serotonin 1f (5-HT1f) receptor,Homo sapiens,126
99. CHEMBL157138,CHEMBL1850,6.061559474,Lysuride,Lisuride,Dopamine D5 receptor,Homo sapiens,274
100. CHEMBL157138,CHEMBL1898,7.495714519,Lysuride,Lisuride,Serotonin 1b (5-HT1b) receptor,Homo sapiens,1236
101. CHEMBL157138,CHEMBL1983,8.169574801,Lysuride,Lisuride,Serotonin 1d (5-HT1d) receptor,Homo sapiens,1251
102. CHEMBL157138,CHEMBL2056,6.318739206,Lysuride,Lisuride,Dopamine D1 receptor,Homo sapiens,895
103. CHEMBL157138,CHEMBL214,7.098680797,Lysuride,Lisuride,Serotonin 1a (5-HT1a) receptor,Homo sapiens,3356
104. CHEMBL157138,CHEMBL219,6.761814213,Lysuride,Lisuride,Dopamine D4 receptor,Homo sapiens,2055
105. CHEMBL157138,CHEMBL224,7.986504353,Lysuride,Lisuride,Serotonin 2a (5-HT2a) receptor,Homo sapiens,2778
106. CHEMBL157138,CHEMBL228,6.90208865,Lysuride,Lisuride,Serotonin transporter,Homo sapiens,4156
107. CHEMBL157138,CHEMBL234,8.440861208,Lysuride,Lisuride,Dopamine D3 receptor,Homo sapiens,3133
108. CHEMBL157138,CHEMBL2490,6.817445204,Lysuride,Lisuride,Serotonin 2a (5-HT2a) receptor,Sus scrofa,99
109. CHEMBL157138,CHEMBL265,6.172266794,Lysuride,Lisuride,Dopamine D1 receptor,Rattus norvegicus,992
110. CHEMBL157138,CHEMBL313,7.585345229,Lysuride,Lisuride,Serotonin transporter,Rattus norvegicus,3527
111. CHEMBL157138,CHEMBL3155,7.317404429,Lysuride,Lisuride,Serotonin 7 (5-HT7) receptor,Homo sapiens,1171
112. CHEMBL157138,CHEMBL322,7.524611215,Lysuride,Lisuride,Serotonin 2a (5-HT2a) receptor,Rattus norvegicus,1648
113. CHEMBL157138,CHEMBL3223,7.670850899,Lysuride,Lisuride,Serotonin 7 (5-HT7) receptor,Rattus norvegicus,365
114. CHEMBL157138,CHEMBL324,7.176562345,Lysuride,Lisuride,Serotonin 2c (5-HT2c) receptor,Rattus norvegicus,575
115. CHEMBL157138,CHEMBL3371,7.757506076,Lysuride,Lisuride,Serotonin 6 (5-HT6) receptor,Homo sapiens,2408
116. CHEMBL157138,CHEMBL339,8.582223883,Lysuride,Lisuride,Dopamine D2 receptor,Rattus norvegicus,4378
117. CHEMBL157138,CHEMBL3426,6.548230465,Lysuride,Lisuride,Serotonin 5a (5-HT5a) receptor,Homo sapiens,466
118. CHEMBL157138,CHEMBL3427,6.817202156,Lysuride,Lisuride,Dopamine D2 receptor,Mus musculus,167
119. CHEMBL157138,CHEMBL3459,7.83025651,Lysuride,Lisuride,Serotonin 1b (5-HT1b) receptor,Rattus norvegicus,397
120. CHEMBL157138,CHEMBL3597,7.212361861,Lysuride,Lisuride,Serotonin 5a (5-HT5a) receptor,Mus musculus,40

121. CHEMBL157138,CHEMBL3666,6.564941495,Lysuride,Lisuride,Serotonin 3a (5-HT3a) receptor,Cavia porcellus,51
122. CHEMBL157138,CHEMBL4105,7.734404367,Lysuride,Lisuride,Serotonin 1d (5-HT1d) receptor,Sus scrofa,127
123. CHEMBL1738797,CHEMBL1875,6.800497634,AF-802,Alectinib,Serotonin 4 (5-HT4) receptor,Homo sapiens,412
124. CHEMBL1738797,CHEMBL1898,6.786469324,AF-802,Alectinib,Serotonin 1b (5-HT1b) receptor,Homo sapiens,1236
125. CHEMBL1738797,CHEMBL1983,7.206493069,AF-802,Alectinib,Serotonin 1d (5-HT1d) receptor,Homo sapiens,1251
126. CHEMBL1738797,CHEMBL214,7.208628833,AF-802,Alectinib,Serotonin 1a (5-HT1a) receptor,Homo sapiens,3356
127. CHEMBL1738797,CHEMBL228,7.419360281,AF-802,Alectinib,Serotonin transporter,Homo sapiens,4156
128. CHEMBL1738797,CHEMBL313,7.528365038,AF-802,Alectinib,Serotonin transporter,Rattus norvegicus,3527
129. CHEMBL1738797,CHEMBL338,6.361870017,AF-802,Alectinib,Dopamine transporter,Rattus norvegicus,2897
130. CHEMBL1738797,CHEMBL3426,5.054740377,AF-802,Alectinib,Serotonin 5a (5-HT5a) receptor,Homo sapiens,466
131. CHEMBL1738797,CHEMBL3459,6.266366611,AF-802,Alectinib,Serotonin 1b (5-HT1b) receptor,Rattus norvegicus,397
132. CHEMBL1738797,CHEMBL3597,6.54328933,AF-802,Alectinib,Serotonin 5a (5-HT5a) receptor,Mus musculus,40
133. CHEMBL1738797,CHEMBL3666,6.531907656,AF-802,Alectinib,Serotonin 3a (5-HT3a) receptor,Cavia porcellus,51
134. CHEMBL1738797,CHEMBL4105,6.582275408,AF-802,Alectinib,Serotonin 1d (5-HT1d) receptor,Sus scrofa,127
135. CHEMBL1738797,CHEMBL4317,6.966702252,AF-802,Alectinib,Serotonin 4 (5-HT4) receptor,Rattus norvegicus,248
136. CHEMBL1738797,CHEMBL5017,6.94418947,AF-802,Alectinib,Serotonin 4 (5-HT4) receptor,Cavia porcellus,273
137. CHEMBL439849,CHEMBL1805,6.946977022,Vilazodone,Vilazodone,Serotonin 1f (5-HT1f) receptor,Homo sapiens,126
138. CHEMBL439849,CHEMBL1833,6.652130658,Vilazodone,Vilazodone,Serotonin 2b (5-HT2b) receptor,Homo sapiens,1238
139. CHEMBL439849,CHEMBL1898,7.175288363,Vilazodone,Vilazodone,Serotonin 1b (5-HT1b) receptor,Homo sapiens,1236
140. CHEMBL439849,CHEMBL1983,7.850689252,Vilazodone,Vilazodone,Serotonin 1d (5-HT1d) receptor,Homo sapiens,1251
141. CHEMBL439849,CHEMBL214,8.513542942,Vilazodone,Vilazodone,Serotonin 1a (5-HT1a) receptor,Homo sapiens,3356
142. CHEMBL439849,CHEMBL219,6.698038231,Vilazodone,Vilazodone,Dopamine D4 receptor,Homo sapiens,2055
143. CHEMBL439849,CHEMBL228,8.400497142,Vilazodone,Vilazodone,Serotonin transporter,Homo sapiens,4156
144. CHEMBL439849,CHEMBL234,7.004902111,Vilazodone,Vilazodone,Dopamine D3 receptor,Homo sapiens,3133
145. CHEMBL439849,CHEMBL313,8.975003346,Vilazodone,Vilazodone,Serotonin transporter,Rattus norvegicus,3527
146. CHEMBL439849,CHEMBL339,6.968002425,Vilazodone,Vilazodone,Dopamine D2 receptor,Rattus norvegicus,4378
147. CHEMBL439849,CHEMBL3426,5.101361752,Vilazodone,Vilazodone,Serotonin 5a (5-HT5a) receptor,Homo sapiens,466
148. CHEMBL439849,CHEMBL3427,6.932459265,Vilazodone,Vilazodone,Dopamine D2 receptor,Mus musculus,167
149. CHEMBL439849,CHEMBL3446,6.279622234,Vilazodone,Vilazodone,Serotonin 2a (5-HT2a) receptor,Bos taurus,128

150. CHEMBL439849,CHEMBL3737,6.984708674,Vilazodone,Vilazodone,Serotonin 1a (5-HT1a) receptor,Mus musculus,70
151. CHEMBL439849,CHEMBL3998,6.808572586,Vilazodone,Vilazodone,Dopamine D2 receptor,Bos taurus,307
152. CHEMBL439849,CHEMBL4105,7.263897444,Vilazodone,Vilazodone,Serotonin 1d (5-HT1d) receptor,Sus scrofa,127
153. CHEMBL439849,CHEMBL4972,6.475030414,Vilazodone,Vilazodone,Serotonin 3a (5-HT3a) receptor,Mus musculus,41
154. CHEMBL45,CHEMBL1805,7.161483517,Vespro,Melatonin,Serotonin 1f (5-HT1f) receptor,Homo sapiens,126
155. CHEMBL45,CHEMBL1833,6.648874773,Vespro,Melatonin,Serotonin 2b (5-HT2b) receptor,Homo sapiens,1238
156. CHEMBL45,CHEMBL1898,7.214843358,Vespro,Melatonin,Serotonin 1b (5-HT1b) receptor,Homo sapiens,1236
157. CHEMBL45,CHEMBL1983,7.620761968,Vespro,Melatonin,Serotonin 1d (5-HT1d) receptor,Homo sapiens,1251
158. CHEMBL45,CHEMBL214,6.884994837,Vespro,Melatonin,Serotonin 1a (5-HT1a) receptor,Homo sapiens,3356
159. CHEMBL45,CHEMBL225,6.544893614,Vespro,Melatonin,Serotonin 2c (5-HT2c) receptor,Homo sapiens,2869
160. CHEMBL45,CHEMBL313,7.291338909,Vespro,Melatonin,Serotonin transporter,Rattus norvegicus,3527
161. CHEMBL45,CHEMBL323,7.402883871,Vespro,Melatonin,Serotonin 2b (5-HT2b) receptor,Rattus norvegicus,213
162. CHEMBL45,CHEMBL3427,6.779739608,Vespro,Melatonin,Dopamine D2 receptor,Mus musculus,167
163. CHEMBL45,CHEMBL3459,6.490416213,Vespro,Melatonin,Serotonin 1b (5-HT1b) receptor,Rattus norvegicus,397
164. CHEMBL45,CHEMBL3666,6.64136081,Vespro,Melatonin,Serotonin 3a (5-HT3a) receptor,Cavia porcellus,51
165. CHEMBL45,CHEMBL3737,6.935311248,Vespro,Melatonin,Serotonin 1a (5-HT1a) receptor,Mus musculus,70
166. CHEMBL45,CHEMBL4105,7.768486387,Vespro,Melatonin,Serotonin 1d (5-HT1d) receptor,Sus scrofa,127
167. CHEMBL493,CHEMBL1805,6.895482077,Parlodel,Bromocriptine,Serotonin 1f (5-HT1f) receptor,Homo sapiens,126
168. CHEMBL493,CHEMBL1850,6.067964521,Parlodel,Bromocriptine,Dopamine D5 receptor,Homo sapiens,274
169. CHEMBL493,CHEMBL1898,7.269444917,Parlodel,Bromocriptine,Serotonin 1b (5-HT1b) receptor,Homo sapiens,1236
170. CHEMBL493,CHEMBL1983,7.429709361,Parlodel,Bromocriptine,Serotonin 1d (5-HT1d) receptor,Homo sapiens,1251
171. CHEMBL493,CHEMBL2056,6.026717231,Parlodel,Bromocriptine,Dopamine D1 receptor,Homo sapiens,895
172. CHEMBL493,CHEMBL214,7.721584288,Parlodel,Bromocriptine,Serotonin 1a (5-HT1a) receptor,Homo sapiens,3356
173. CHEMBL493,CHEMBL219,6.705084046,Parlodel,Bromocriptine,Dopamine D4 receptor,Homo sapiens,2055
174. CHEMBL493,CHEMBL224,7.511922024,Parlodel,Bromocriptine,Serotonin 2a (5-HT2a) receptor,Homo sapiens,2778
175. CHEMBL493,CHEMBL225,6.805702684,Parlodel,Bromocriptine,Serotonin 2c (5-HT2c) receptor,Homo sapiens,2869
176. CHEMBL493,CHEMBL228,7.077181647,Parlodel,Bromocriptine,Serotonin transporter,Homo sapiens,4156
177. CHEMBL493,CHEMBL234,8.740090118,Parlodel,Bromocriptine,Dopamine D3 receptor,Homo sapiens,3133
178. CHEMBL493,CHEMBL2490,6.951517225,Parlodel,Bromocriptine,Serotonin 2a (5-HT2a) receptor,Sus scrofa,99

179. CHEMBL493,CHEMBL313,7.320674107,Parlodel,Bromocriptine,Serotonin transporter,Rattus norvegicus,3527
180. CHEMBL493,CHEMBL3155,7.286448453,Parlodel,Bromocriptine,Serotonin 7 (5-HT7) receptor,Homo sapiens,1171
181. CHEMBL493,CHEMBL322,7.617506562,Parlodel,Bromocriptine,Serotonin 2a (5-HT2a) receptor,Rattus norvegicus,1648
182. CHEMBL493,CHEMBL3223,7.688277222,Parlodel,Bromocriptine,Serotonin 7 (5-HT7) receptor,Rattus norvegicus,365
183. CHEMBL493,CHEMBL324,7.437469531,Parlodel,Bromocriptine,Serotonin 2c (5-HT2c) receptor,Rattus norvegicus,575
184. CHEMBL493,CHEMBL3371,7.62028838,Parlodel,Bromocriptine,Serotonin 6 (5-HT6) receptor,Homo sapiens,2408
185. CHEMBL493,CHEMBL339,7.797872912,Parlodel,Bromocriptine,Dopamine D2 receptor,Rattus norvegicus,4378
186. CHEMBL493,CHEMBL3426,6.989271881,Parlodel,Bromocriptine,Serotonin 5a (5-HT5a) receptor,Homo sapiens,466
187. CHEMBL493,CHEMBL3427,6.641548349,Parlodel,Bromocriptine,Dopamine D2 receptor,Mus musculus,167
188. CHEMBL493,CHEMBL3459,7.546512792,Parlodel,Bromocriptine,Serotonin 1b (5-HT1b) receptor,Rattus norvegicus,397
189. CHEMBL493,CHEMBL3597,7.504955266,Parlodel,Bromocriptine,Serotonin 5a (5-HT5a) receptor,Mus musculus,40
190. CHEMBL493,CHEMBL3666,6.344296776,Parlodel,Bromocriptine,Serotonin 3a (5-HT3a) receptor,Cavia porcellus,51
191. CHEMBL493,CHEMBL4105,6.934012684,Parlodel,Bromocriptine,Serotonin 1d (5-HT1d) receptor,Sus scrofa,127
192. CHEMBL493,CHEMBL4317,7.033321222,Parlodel,Bromocriptine,Serotonin 4 (5-HT4) receptor,Rattus norvegicus,248
193. CHEMBL502835,CHEMBL1850,6.163045514,Vargatef,Nintedanib,Dopamine D5 receptor,Homo sapiens,274
194. CHEMBL502835,CHEMBL2056,6.04438833,Vargatef,Nintedanib,Dopamine D1 receptor,Homo sapiens,895
195. CHEMBL502835,CHEMBL214,6.715580061,Vargatef,Nintedanib,Serotonin 1a (5-HT1a) receptor,Homo sapiens,3356
196. CHEMBL502835,CHEMBL2490,7.1288118,Vargatef,Nintedanib,Serotonin 2a (5-HT2a) receptor,Sus scrofa,99
197. CHEMBL502835,CHEMBL313,7.086542977,Vargatef,Nintedanib,Serotonin transporter,Rattus norvegicus,3527
198. CHEMBL502835,CHEMBL3597,6.548714861,Vargatef,Nintedanib,Serotonin 5a (5-HT5a) receptor,Mus musculus,40
199. CHEMBL502835,CHEMBL3666,6.404754196,Vargatef,Nintedanib,Serotonin 3a (5-HT3a) receptor,Cavia porcellus,51
200. CHEMBL502835,CHEMBL4105,6.461116463,Vargatef,Nintedanib,Serotonin 1d (5-HT1d) receptor,Sus scrofa,127
201. CHEMBL502835,CHEMBL4317,6.959182715,Vargatef,Nintedanib,Serotonin 4 (5-HT4) receptor,Rattus norvegicus,248
202. CHEMBL502835,CHEMBL5067,5.802860045,Vargatef,Nintedanib,Dopamine D1 receptor,Sus scrofa,240
203. CHEMBL56564,CHEMBL1805,7.120643415,Tropisetron,Tropisetron,Serotonin 1f (5-HT1f) receptor,Homo sapiens,126
204. CHEMBL56564,CHEMBL1898,6.652799389,Tropisetron,Tropisetron,Serotonin 1b (5-HT1b) receptor,Homo sapiens,1236
205. CHEMBL56564,CHEMBL1899,7.58982091,Tropisetron,Tropisetron,Serotonin 3a (5-HT3a) receptor,Homo sapiens,477
206. CHEMBL56564,CHEMBL1983,7.523089092,Tropisetron,Tropisetron,Serotonin 1d (5-HT1d) receptor,Homo sapiens,1251
207. CHEMBL56564,CHEMBL214,7.197181732,Tropisetron,Tropisetron,Serotonin 1a (5-HT1a) receptor,Homo sapiens,3356

208. CHEMBL56564,CHEMBL228,6.891886669,Tropisetron,Tropisetron,Serotonin transporter,Homo sapiens,4156
209. CHEMBL56564,CHEMBL2490,6.819426575,Tropisetron,Tropisetron,Serotonin 2a (5-HT2a) receptor,Sus scrofa,99
210. CHEMBL56564,CHEMBL313,7.245124224,Tropisetron,Tropisetron,Serotonin transporter,Rattus norvegicus,3527
211. CHEMBL56564,CHEMBL3371,6.947741034,Tropisetron,Tropisetron,Serotonin 6 (5-HT6) receptor,Homo sapiens,2408
212. CHEMBL56564,CHEMBL3459,6.445404525,Tropisetron,Tropisetron,Serotonin 1b (5-HT1b) receptor,Rattus norvegicus,397
213. CHEMBL56564,CHEMBL3666,6.978388917,Tropisetron,Tropisetron,Serotonin 3a (5-HT3a) receptor,Cavia porcellus,51
214. CHEMBL56564,CHEMBL4105,7.339656077,Tropisetron,Tropisetron,Serotonin 1d (5-HT1d) receptor,Sus scrofa,127
215. CHEMBL56564,CHEMBL5017,6.825535838,Tropisetron,Tropisetron,Serotonin 4 (5-HT4) receptor,Cavia porcellus,273
216. CHEMBL56564,CHEMBL5453,6.177531111,Tropisetron,Tropisetron,Serotonin transporter,Macaca mulatta,67
217. CHEMBL76370,CHEMBL1805,6.8860486,Zelmac,Tegaserod,Serotonin 1f (5-HT1f) receptor,Homo sapiens,126
218. CHEMBL76370,CHEMBL1833,6.631538191,Zelmac,Tegaserod,Serotonin 2b (5-HT2b) receptor,Homo sapiens,1238
219. CHEMBL76370,CHEMBL1898,6.792082958,Zelmac,Tegaserod,Serotonin 1b (5-HT1b) receptor,Homo sapiens,1236
220. CHEMBL76370,CHEMBL225,6.988031088,Zelmac,Tegaserod,Serotonin 2c (5-HT2c) receptor,Homo sapiens,2869
221. CHEMBL76370,CHEMBL313,7.367280212,Zelmac,Tegaserod,Serotonin transporter,Rattus norvegicus,3527
222. CHEMBL76370,CHEMBL323,7.279111879,Zelmac,Tegaserod,Serotonin 2b (5-HT2b) receptor,Rattus norvegicus,213
223. CHEMBL76370,CHEMBL3426,5.238982323,Zelmac,Tegaserod,Serotonin 5a (5-HT5a) receptor,Homo sapiens,466
224. CHEMBL76370,CHEMBL3427,6.606960205,Zelmac,Tegaserod,Dopamine D2 receptor,Mus musculus,167
225. CHEMBL76370,CHEMBL3459,6.640007596,Zelmac,Tegaserod,Serotonin 1b (5-HT1b) receptor,Rattus norvegicus,397
226. CHEMBL76370,CHEMBL3666,6.563390966,Zelmac,Tegaserod,Serotonin 3a (5-HT3a) receptor,Cavia porcellus,51
227. CHEMBL76370,CHEMBL3737,6.804286024,Zelmac,Tegaserod,Serotonin 1a (5-HT1a) receptor,Mus musculus,70
228. CHEMBL76370,CHEMBL4105,7.312055859,Zelmac,Tegaserod,Serotonin 1d (5-HT1d) receptor,Sus scrofa,127
229. CHEMBL76370,CHEMBL4317,7.181530849,Zelmac,Tegaserod,Serotonin 4 (5-HT4) receptor,Rattus norvegicus,248
230. CHEMBL76370,CHEMBL5017,7.484500086,Zelmac,Tegaserod,Serotonin 4 (5-HT4) receptor,Cavia porcellus,273
231. CHEMBL905,CHEMBL1805,7.0784031,MK-462,Rizatriptan,Serotonin 1f (5-HT1f) receptor,Homo sapiens,126
232. CHEMBL905,CHEMBL1983,8.06345622,MK-462,Rizatriptan,Serotonin 1d (5-HT1d) receptor,Homo sapiens,1251
233. CHEMBL905,CHEMBL214,6.674010619,MK-462,Rizatriptan,Serotonin 1a (5-HT1a) receptor,Homo sapiens,3356
234. CHEMBL905,CHEMBL228,7.0961303,MK-462,Rizatriptan,Serotonin transporter,Homo sapiens,4156
235. CHEMBL905,CHEMBL2490,7.370009895,MK-462,Rizatriptan,Serotonin 2a (5-HT2a) receptor,Sus scrofa,99
236. CHEMBL905,CHEMBL3102,4.682608787,MK-462,Rizatriptan,Dopamine beta-hydroxylase,Homo sapiens,32

237. CHEMBL905,CHEMBL313,7.240521883,MK-462,Rizatriptan,Serotonin transporter,Rattus norvegicus,3527
238. CHEMBL905,CHEMBL3426,5.263271106,MK-462,Rizatriptan,Serotonin 5a (5-HT5a) receptor,Homo sapiens,466
239. CHEMBL905,CHEMBL3427,6.705909653,MK-462,Rizatriptan,Dopamine D2 receptor,Mus musculus,167
240. CHEMBL905,CHEMBL3459,6.361374468,MK-462,Rizatriptan,Serotonin 1b (5-HT1b) receptor,Rattus norvegicus,397
241. CHEMBL905,CHEMBL3666,6.498689799,MK-462,Rizatriptan,Serotonin 3a (5-HT3a) receptor,Cavia porcellus,51
242. CHEMBL905,CHEMBL3737,6.794487705,MK-462,Rizatriptan,Serotonin 1a (5-HT1a) receptor,Mus musculus,70
243. CHEMBL905,CHEMBL4105,7.324676495,MK-462,Rizatriptan,Serotonin 1d (5-HT1d) receptor,Sus scrofa,127
244. CHEMBL905,CHEMBL5377,4.475752434,MK-462,Rizatriptan,Serotonin 2a (5-HT2a) receptor,Mus musculus,68
245. CHEMBL905,CHEMBL5450,6.86476544,MK-462,Rizatriptan,Serotonin 1d (5-HT1d) receptor,Rattus norvegicus,48
